# Preclinical insights into the gut‐skeletal muscle axis in chronic gastrointestinal diseases

**DOI:** 10.1111/jcmm.15554

**Published:** 2020-07-06

**Authors:** Luise Ehlers, Karen Bannert, Sarah Rohde, Peggy Berlin, Johannes Reiner, Mats Wiese, Julia Doller, Markus M. Lerch, Ali A. Aghdassi, Fatuma Meyer, Luzia Valentini, Ottavia Agrifoglio, Cornelia C. Metges, Georg Lamprecht, Robert Jaster

**Affiliations:** ^1^ Department of Medicine II Division of Gastroenterology Rostock University Medical Center Rostock Germany; ^2^ Department of Medicine A University Medicine Greifswald Greifswald Germany; ^3^ Department of Agriculture and Food Sciences Neubrandenburg Institute of Evidence‐Based Nutrition (NIED) University of Applied Sciences Neubrandenburg Neubrandenburg Germany; ^4^ Institute of Nutritional Physiology 'Oskar Kellner' Leibniz Institute for Farm Animal Biology (FBN) Dummerstorf Germany

**Keywords:** chronic liver disease, chronic pancreatitis, inflammatory bowel disease, malnutrition, pancreatic cancer, sarcopenia

## Abstract

Muscle wasting represents a constant pathological feature of common chronic gastrointestinal diseases, including liver cirrhosis (LC), inflammatory bowel diseases (IBD), chronic pancreatitis (CP) and pancreatic cancer (PC), and is associated with increased morbidity and mortality. Recent clinical and experimental studies point to the existence of a gut‐skeletal muscle axis that is constituted by specific gut‐derived mediators which activate pro‐ and anti‐sarcopenic signalling pathways in skeletal muscle cells. A pathophysiological link between both organs is also provided by low‐grade systemic inflammation. Animal models of LC, IBD, CP and PC represent an important resource for mechanistic and preclinical studies on disease‐associated muscle wasting. They are also required to test and validate specific anti‐sarcopenic therapies prior to clinical application. In this article, we review frequently used rodent models of muscle wasting in the context of chronic gastrointestinal diseases, survey their specific advantages and limitations and discuss possibilities for further research activities in the field. We conclude that animal models of LC‐, IBD‐ and PC‐associated sarcopenia are an essential supplement to clinical studies because they may provide additional mechanistic insights and help to identify molecular targets for therapeutic interventions in humans.

## 1 INTRODUCTION

In developed countries, muscle wasting occurs largely in the setting of disease. It often accompanies chronic gastrointestinal diseases such as liver cirrhosis (LC), inflammatory bowel diseases (IBD) or pancreatic cancer (PC) and is among the defining criteria for sarcopenia, cachexia and disease‐related malnutrition (DRM).^1^, ^2^ Systemic pro‐inflammatory conditions often cause these catabolic processes^3^ as seen in metastatic cancer, sepsis, tuberculosis or HIV. Sarcopenia is present in up to 70% of patients with LC,^4^ approximately 17% of patients with chronic pancreatitis (CP) and ranges from 30% to 65% in patients with PC.^5^ In German hospitals and in the Western world in general, every fourth patient is malnourished with a particularly high prevalence of >30% in gastroenterology.^6^ The prevalence of malnutrition is estimated to be between 20% and 60% in LC patients, and up to 75% and 62% in patients with Crohn's disease and ulcerative colitis, respectively.^7^ A recent large study from Spain reported a more conservative estimate of 16% prevalence of malnutrition in IBD patients.^8^

Sarcopenia, that is muscle failure, is defined as a muscle disease rooted in adverse muscle changes that accrue across a lifetime.^1^ The loss of muscle mass and function in sarcopenia is often associated with physical disability, poor quality of life, increased hospital admissions and increased mortality.^9^ Sarcopenia is common among the elderly and was initially considered being a geriatric syndrome. However, its definition has been expanded to include its development in younger patients due to malnutrition and also the existence of chronic disease and inflammation.^10^ According to the current consensus on definition and diagnosis of sarcopenia, low muscle strength is the key characteristic, low muscle quantity and quality are used to confirm diagnosis and poor physical performance indicates severe sarcopenia.^1^ Malnutrition may not only be caused by compromised intake or assimilation of nutrients but also by concomitant diseases specifying this type as DRM.^2^ Pathophysiologically, DRM is often triggered by a sustained disease‐specific inflammatory response of mild to moderate degree. The chronic state accompanied by a milder inflammatory response is also called cachexia, although cachexia is often incorrectly perceived as end‐stage malnutrition.^2^ The chronic inflammation contributes to malnutrition through associated anorexia and decreased food intake as well as an altered metabolism with elevation of resting energy expenditure and increased muscle catabolism.^2^, ^11^ Consequently, DRM leads to an altered body composition manifesting as a decrease in muscle mass and thereby is associated with adverse functional and clinical outcomes.^11^ Malnutrition and sarcopenia often manifest clinically through a combination of decreased nutrient intake, inflammation and decreased body weight, along with a decrease in muscle mass, strength and/or physical function.^6^, ^10^

In liver cirrhosis, malnutrition plays a central role in muscle loss, which is not restricted to a reduced energy intake. Malnourished LC patients were also shown to have an increase of the intestinal permeability^12^ and changes in the gut microbiota.^13^ It is assumed that a microbial translocation and the consecutive inflammation contribute to sarcopenia in LC patients.^4^ Muscle wasting is associated with complications like infections, ascites or hepatic encephalopathy and with an impaired prognosis and higher mortality.^14^ Besides malnutrition, an altered lipid and carbohydrate metabolism, inhibition of muscle growth, inflammation and inactivity contribute to muscle loss in LC.^15^ Due to the multifactorial aetiology, muscle loss in LC is not adequately treated by nutrition and physical activity alone.^15^ In patients with LC, the transjugular intrahepatic portosystemic shunt (TIPS) is widely used to treat portal hypertension, especially in the management of refractory variceal bleeding and refractory ascites. After successful treatment with TIPS, body weight gain and improvement in the nutritional status have been widely reported. In malnourished LC patients with hypermetabolism who underwent TIPS placement, an enhancement in the body cell and muscle mass and thereby an improvement in the body composition was described.^16^ Further, TIPS was shown to reverse sarcopenia whereas failure to improve muscle area after TIPS was accompanied by higher mortality.^17^ Other strategies to minimize or reverse muscle loss include branched chain amino acid, L‐ornithine and L‐aspartate supplementation as well as Rifaximin administration.^18^, ^19^, ^20^ However, due to the multifactorial aetiology and the selection of the best possible therapy, it is important to understand the complex metabolic and hormonal changes associated with muscle wasting in LC.

Malnutrition is also highly prevalent among patients with inflammatory bowel disease, especially with Crohn's disease,^21^ and occurs in active as well as remission phases of the disease. Further, it has been shown that malnourished patients more often suffer from sarcopenia as well.^7^, ^22^ It is assumed that the process of muscle wasting is reversible in IBD patients when the underlying disease is treated.^23^ However, muscle loss in IBD is common, negatively impacts various clinical outcomes and is associated with increased hospitalizations, disease flares, the need for surgery and post‐operative complications.^8^ Recent studies have indicated that low muscle mass is an independent predictor of the surgical outcome and that sarcopenic IBD patients who require surgery are often in need of increased post‐operative care.^22^, ^23^ It was shown that the prevalence of muscle wasting is related to a deficiency of vitamin D, linking the muscular failure to the nutritional status.^24^ Further molecular mechanisms of muscle wasting in IBD remain to be elucidated.

In chronic pancreatitis, repeated episodes of inflammation induce fibrosis,^25^ eventually resulting in the loss of pancreatic exocrine and endocrine function. Impairment of exocrine pancreatic function is also the single most important factor regulating intestinal microbiota composition and thus predisposing to gut dysbiosis.^26^ Malnutrition and sarcopenia are frequent complications of CP. Besides pancreatic exocrine insufficiency, small intestinal bacterial overgrowth, pain‐induced anorexia and ongoing alcohol and nicotine consumption present presumed risk factors.^27^, ^28^ An elevated resting energy expenditure, found in 30%‐50% of patients, may further contribute to loss of muscle mass and function.^29^ For malnutrition and sarcopenia alike, the use of inconsistent definitions and diagnostic modalities makes accurate prevalence estimation difficult. However, reported prevalences of 8%‐39% and 17%‐52% for malnutrition based on body mass index and sarcopenia, respectively, indicate that both conditions are common manifestations in CP.^27^, ^30^, ^31^ Because both malnutrition and sarcopenia have been associated with adverse health‐related outcomes and increased mortality,^27^, ^32^ treatment can be considered mandatory. Although loss of muscle mass is an inherent overlap between the two conditions, it is still elusive whether identical therapy should be applied. Unfortunately, understanding of the underlying pathophysiology is still limited. Advanced insights into interrelations between malnutrition, sarcopenia, and CP therefore hold potential to benefit future therapeutic options. Pancreatic cancer (PC) represents the fourth leading cause of cancer‐related mortality.^33^ Malnutrition is a common phenomenon in PC patients and negatively influences the quality of life, length of hospital stay and survival.^34^, ^35^ Specifically, failure of exocrine and endocrine functions can lead to malnutrition, resulting in a catabolic state.^35^ Sarcopenia in PC patients is associated with a worse prognosis,^5^ and preoperative sarcopenia was found to be associated with a prolonged hospital stay after pancreatic surgery.^36^ It has been suggested that muscle loss is influenced by an imbalance of anabolic and catabolic pathways as well as systemic inflammation,^37^ but the underlying molecular mechanisms are poorly understood yet.

## 2 THE GUT‐SKELETAL MUSCLE AXIS

A growing body of evidence suggests that gastrointestinal organs and skeletal muscles are directly linked to each other by a network of hormones, metabolites and mediators that together form the so‐called gut‐skeletal muscle axis.^38^, ^39^ In this chapter, we provide a short overview of the current knowledge in this field.

The gastrointestinal tract constitutes an important source of hormones that directly and indirectly affect morphology and function of skeletal muscles. Gut‐derived hormones with potential anti‐sarcopenic effects are glucagon‐like peptide‐1 (GLP‐1) and −2 (GLP‐2) as well as ghrelin. GLP‐1 agonists, which are widely used for the treatment of diabetes mellitus, have recently been proposed to ameliorate muscle wasting by suppressing myostatin and muscle atrophic factors (such as *atrogin‐1* and muscle *RING‐finger protein‐1* (*MuRF‐1*)), and enhancing myogenic factors including *MyoG* and *MyoD*.^40^ Pharmacologic GLP‐2‐stimulation promotes gut‐specific growth, enhances intestinal epithelial function and alleviates malnutrition, likely secondary contributes to the gut‐specific pro‐absorptive effects.^41^ Similarly, ghrelin has been suggested to exert protective effects on fasting‐induced muscle atrophy in ageing mice through an enhanced expression of myogenic genes and decreased expression of degradation genes.^42^ The effects of these hormones on muscle wasting associated with chronic gastrointestinal diseases have not been studied yet.

A plethora of nutritional components also plays specific roles in the maintenance and regulation of muscle metabolism. These substances include, but are not restricted to, individual proteins and amino acids,^43^, ^44^ vitamins (especially, vitamin D^45^) and various lipid components, such as n‐3 polyunsaturated fatty acids.^46^, ^47^ Since a detailed survey of this aspect is beyond the scope of this paper, we kindly refer the reader to a more dedicated recent review in the field.^39^

The gut microbiome influences and modulates the gut‐skeletal muscle axis through a variety of microbiota‐derived metabolites and other molecules. Prominent examples include short‐chain fatty acids (eg butyrate), secondary bile acids, water‐soluble B‐vitamins, the amino acid tryptophan, polyphenols and antioxidants (eg urolithines) as well as pathogen‐associated molecular patterns, which, under pathophysiological conditions, trigger inflammatory responses by interacting with pattern‐recognition receptors on cells of the innate immune system.^38^, ^39^

Disease‐associated imbalances of the gut‐skeletal muscle axis are frequently accompanied, mediated and/or enhanced by a low‐grade systemic inflammation, characterized by increased levels of pro‐inflammatory cytokines (such as tumour necrosis factor‐α (TNF‐α) and interleukin‐6 (IL‐6)), elevated circulating lipopolysaccharide (eg in gastrointestinal diseases) and a predominance of anti‐ vs pro‐myogenic mediators (eg myostatin vs insulin‐like growth factor‐1 (IGF‐1)).^39^, ^48^

In skeletal muscle cells, evolutionary conserved signalling cascades constitute the point of convergence for the action of many mediators mentioned above. To this end, the following signal transduction pathways have emerged as particularly important with respect to the induction or inhibition of muscle wasting in the context of gastrointestinal diseases: myostatin, a member of the transforming growth factor‐β (TGF‐β) protein family, exerts its inhibitory effects on skeletal muscle growth through its binding to the activin type 2B receptor and activation of mothers against decapentaplegic homolog (Smad) 2 and 3 transcription factors.^49^ Subsequently, inhibition of the phosphatidylinositol 3‐kinase (PI 3‐K)/protein kinase B (AKT)/mammalian target of rapamycin (mTOR) pathway results in diminished protein synthesis and muscle atrophy.^50^ Two major antagonists of myostatin are follistatin and IGF‐1, which interfere with its effects by direct interaction (follistatin),^51^ or at the level of PI 3‐K/AKT/mTOR through a (re)‐activation of the signalling cascade.^52^

Pro‐inflammatory mediators such as TNF‐α, but also hyperammonemia, activate nuclear factor ‘kappa‐light‐chain‐enhancer’ of activated B cells (NF‐κB) signalling and induce, in part mediated by myostatin, downstream target genes including *MuRF‐1* and *atrogin‐1* (*MAFbx*) that promote muscle protein degradation through the proteasomal pathway.^53^, ^54^ Protein degradation can also be triggered by increased levels of reactive oxygen species and subsequent activation of the transcription factor FoxO.^55^

Additional molecular processes that have been implicated in disease‐associated muscle wasting are enhancement of autophagy (eg via LC3/p62‐SQSTM1)^54^, ^56^ and induction of mitochondrial dysfunction.^39^ These pathophysiological processes may also result in cell death by apoptosis (rather than necrosis), which contributes to the progress of sarcopenia.^56^

## 3 ANIMAL MODELS OF MUSCLE WASTING IN CHRONIC GASTROINTESTINAL DISEASES

Animal models of LC, IBD and PC represent an important resource for preclinical studies on disease‐associated muscle wasting and have provided significant pathomechanistic insights. In this chapter, we review frequently used animal models of gastrointestinal diseases associated with muscle wasting and focus on their specific advantages but also their limitations.

### 3.1 Rodent models of chronic liver disease‐associated muscle wasting

The most widely used rodent models of muscle wasting in the context of chronic liver disease comprise the models of portocaval shunts (PS), bile duct ligation (BDL) and carbon tetrachloride (CCl_4_) administration in rats or mice (Table 1).

**Table 1:** Rodent models of chronic liver disease‐associated muscle wasting and their key features (for references, see text)

Feature	Model		
	Portocaval shunt	Bile duct ligation	CCl_4_ application
Technique	Surgery: end‐to side portocaval anastomosis	Surgery: ligation and transection of the common bile duct	Repeated application by injection or oral gavage
Species	Rat	Rat, mouse	Rat, mouse
Injury	Bypass of portal blood flow	Cholestasis, liver fibrosis/cirrhosis	Toxic hepatopathy, liver fibrosis/cirrhosis
Skeletal muscle morphology	Loss of weight; atrophy Impaired satellite cell proliferation and differentiation	Loss of weight Reduction of total fibres and cross‐sectional area	Loss of weight Reduction of total fibres and cross‐sectional area
Skeletal muscle function	Reduced grip strength	Affected kinetic properties (slowing down) Decrease of muscle force and resistance to fatigue	Slowing down of the kinetic properties
Key biochemical abnormalities	Hyperammonemia Uninhibited proteolysis Decreased muscle protein synthesis	Decreased muscle protein synthesis Increased protein degradation	Enhanced proteolysis and protein degradation Increase of pro‐inflammatory mediators (IL‐6, TNF‐α)
Molecular pathways in muscle dysfunction	Activation of NF‐κB Increase of myostatin	Increase of myostatin Inhibition of PI 3‐K/Akt/mTOR Triggering of autophagy pathways	Activation of NF‐κB and of the ubiquitin‐proteasome proteolytic pathway Induction of IL‐6 expression
Relations to human disease	TIPS patients with cirrhosis	Cholestatic liver diseases Cirrhosis and extrahepatic complications	Toxic hepatopathies Cirrhosis and extrahepatic complications
Limitations for studies on myopenia	Lack of liver cirrhosis and systemic inflammation	Severity of the model with complete cholestasis Uncertain role of inflammation	Low relevance of CCl4 in human cirrhosis Concerns regarding reproducibility and mortality

The PS model has been established in rats^57^, ^58^, ^59^ and shares with the BDL model the need for microsurgical skills and equipment. In 6‐weeks‐old male Sprague‐Dawley rats, a portocaval shunt, within 4 weeks, leads to nutritional and metabolic changes similar to those that occur in human cirrhosis. Notably, it also causes muscle wasting and skeletal muscle atrophy, which are accompanied by lower skeletal muscle mass and grip strength.^57^ Muscle wasting in this model has been shown to be the consequence of impaired satellite cell proliferation and differentiation, and linked to lower IGF‐1/IGF‐1‐receptor expression as well as higher mRNA and protein levels of myostatin, activin type 2B receptor and cyclin‐dependent kinase inhibitor p21 (CDKN1A).^58^ Together, these findings are compatible with the concept of an uninhibited proteolysis and a reduced rate of muscle protein synthesis. Interestingly, application of follistatin, a potent myostatin antagonist, was sufficient to largely reverse muscle wasting in this model.^59^ As a key mechanistic factor in PS‐triggered myopenia, hyperammonemia has been proposed.^53^, ^60^ Specifically, hyperammonemia was found to stimulate myostatin expression in a NF‐κB‐dependent manner^53^ and to activate autophagy in skeletal muscle.^60^ Autophagy could be particularly relevant for degradation of nitrated muscle proteins, which are increased both in patients and rats with LC.^60^

Transjugular intrahepatic portosystemic shunt improves survival in well selected LC patients.^61^ However, TIPS may even worsen liver function^62^ and, conversely, can be associated with muscle wasting.^59^ Furthermore, hyperammonemia, as described in the PS model, is also frequently observed in patients with TIPS and may cause complications such as enhanced hepatic encephalopathy. Hyperammonemia is one of the major causes of muscular growth inhibition.^53^, ^60^ Taken together, the PS model can be considered as clinically relevant in the special context of TIPS‐associated muscle wasting, where it represents a versatile tool to gain further insights into mechanisms of proteolysis and reduced protein synthesis. Limitations of the model include the lack of histological LC, as well as the virtual absence of hepatic/systemic inflammation and cellular injury responses.^59^

First studies in bile duct‐ligated rodents go back as far as 1932, when Cameron and Oakley^63^ introduced the technique in a rat model. In response to biliary obstruction for 15 days or more, rats develop progressive cirrhosis associated with ascites.^64^ In the meantime, the BDL model has been widely used for studies in the field of cholestatic liver disease, liver fibrosis and cirrhosis, and shown to be applicable in mice as well.^65^ Of note, there are significant species‐ and strain‐specific differences with respect to the time course and the severity of the hepatic injury. In C57BL/6 mice, an early phase of biliary infarction is followed by tissue inflammation, proliferation of hepatocytes and cholangiocytes (week 1) and accumulation of collagen (week 2), resulting in stabilized liver fibrosis thereafter. Development of LC in bile duct‐ligated mice is accompanied by progressive skeletal muscle wasting.^54^, ^66^ Specifically, a loss of skeletal muscle weight (m. quadriceps, m. gastrocnemius), a reduction of total fibres and the cross‐sectional area (m. quadriceps), a slowing down of the kinetic properties and a decrease of muscle force and resistance to fatigue (m. extensor digitorum longus) were observed.^54^ Furthermore, in BDL rat models 6 weeks after surgery, protein synthesis was decreased in gastrocnemius muscle but not in other organs.^67^

To this end, the molecular basis of myopenia in bile duct‐ligated rodents remains to be fully elucidated. As one key mechanism, increased degradation of muscle proteins through the ubiquitin‐proteasome pathway has been proposed and locally acting TNF‐α implicated in the mediation of these effects.^66^ In FVB mice, decreased protein synthesis, in addition to increased protein degradation, has been suggested as the principal cause of skeletal muscle atrophy. Blocking of protein synthesis could be attributed to increased levels of myostatin, which inhibited the PI 3‐K/Akt/mTOR pathway. In addition, myostatin might trigger autophagy, thereby further enhancing the development of muscle atrophy in bile duct‐ligated FVB mice.^54^ In the mouse model, neither enhanced muscle tissue levels of TNF‐α nor of IL‐6 were observed.^54^ Furthermore, to the best of our knowledge, no evidence for a pivotal role of systemic inflammation in myopenia of bile duct‐ligated rodents has been reported, rendering the model less suitable for the analysis of this important aspect of the human disease. Additionally, the relatively short time course to disease onset is in contrast to the rather slow progression of chronic liver disease in humans.^68^ The advantages of this model are the long availability, the therefore well‐studied structural and functional changes which are induced by BDL, as well as its reproducibility.^68^

The CCl_4_ model is considered prototypical for rodent models of LC that rely on the application of hepatotoxins. Induction of LC requires the repeated application of CCl_4_ over a period of 2‐3 months (eg twice a week). Susceptibility to CCl_4_ liver injury and fibrogenesis in mice largely depends on the genetic background,^69^ with BALB/c as the most sensitive strain.^70^ CCl_4_ exerts consistent hepatotoxicity through its reactive metabolites generated by cytochrome P‐450 enzymes, primarily CYP2E1, expressed in perivenular hepatocytes.^71^ Studies on cirrhosis‐associated muscle wasting have been performed both in mice and rat.^54^, ^72^ The CCl_4_ model has been suggested to closely resemble the biochemical, histological and haemodynamic alterations observed in human patients, including development of portal hypertension, ascites, collateral venous channels and low branched chain/aromatic amino acids plasma ratio.^73^ Still, as a hepatotoxin, CCl_4_ remains a model substance with limited relevance for human LC. Other drawbacks include the unsatisfactory reproducibility and associated mortality of CCl_4_ administration.^71^

Morphological and functional alterations of skeletal muscles in CCl_4_‐treated mice resemble the changes observed in bile duct‐ligated animals, except that muscle force and time to fatigue remain unaffected. At the molecular level, however, major mechanistic differences were observed: in contrast to the BDL model, in mice with CCl_4_‐induced LC, a selective upregulation of TNF‐α and IL‐6 in muscle tissue was detected. Both cytokines activate NF‐κB, which in turn induces transcription of components of the ubiquitin‐proteasome proteolytic pathway such as *atrogin‐1* and *MuRF‐1*, thus triggering protein degradation. In addition, NF‐κB further enhances expression of IL‐6, thereby creating a vicious cycle of inflammation and muscle protein degradation.^54^

In summary, each of the three models described above is characterized by a unique pathophysiology that resembles some but not all aspects of human chronic liver disease. Inhibition of protein synthesis and/or enhancement of protein degradation represent common features that can be studied in each of the models, although the underlying molecular mechanisms are to some degree variable. The two surgical models may not be applicable to the analysis of inflammation‐associated processes of muscle wasting, whereas the toxicity and variable reproducibility of its effects limit the use of CCl_4_. Table 1 summarizes key features of the three models.

All three rodent models of chronic liver disease lead to body weight loss, indicative of malnutrition in these animals. Malnutrition has not been investigated systematically in these models and most of the literature was published before the year 2000. To the best of our knowledge, the gut‐skeletal muscle axis in rodent models of chronic liver disease has not been surveyed yet. Intriguingly, CCl_4_‐induced liver cirrhosis can be improved by oral administration of *S boulardii*, which reduces the intestinal permeability and modulates the gut microbiome composition.^74^ Also, protective effects against bacterial antigen translocation via oral application of *B pseudocatenulatum* CECT7765 have been demonstrated in experimental cirrhosis.^75^ Effects on the underlying disease most likely also have an impact on the disease‐related muscle wasting.

### 3.2 Models of muscle wasting in inflammatory bowel diseases

Even in patients with Crohn's disease in clinical remission, prevalence of muscle wasting is as high as 60%.^76^ Despite this high clinical relevance, mechanistic in‐depth studies in animal models of IBD are rare.

Both in mice and rats, intrarectal administration of trinitrobenzene sulphonic acid (TNBS) induces a transmural and chronic colonic inflammation similar to that observed in patients with Crohn's disease.^77^, ^78^ TNBS‐induced colitis is associated with muscle wasting, as indicated by the reduction of both skeletal muscle mass and protein content. As a central mechanism, increased protein degradation through proteasome activation and enhanced expression of the muscle‐specific atrogenes *MuRF‐1* and *atrogin‐1* have been proposed, whereas the rate of muscle protein synthesis remained unchanged. Furthermore, TNF‐α, IL‐6 and NOS2 mRNA content of both liver and skeletal muscle are increased in TNBS‐treated mice, and plasma TNF‐α and IL‐6 concentrations were found to be elevated as well.^78^ The model therefore nicely reflects both systemic and local inflammatory processes, which are thought also to play a key role in muscle wasting in patients.

Application of dextran sulphate sodium (DSS) to mice through drinking water induces an intestinal inflammation that histologically resembles human ulcerative colitis.^77^, ^79^ Mice with DSS colitis develop severe muscle wasting, as indicated by reduced skeletal muscle weight (m. quadriceps, m. gastrocnemius), a decrease in muscle fibre size, affecting both type 1 and 2 fibres and diminished muscle protein content. Furthermore, increased mRNA expression of the E3 ligases *MuRF‐1* and *Atrogin1/MAFbx* was observed, implicating enhanced protein degradation into the pathogenesis of muscle atrophy.^80^ DSS colitis has therefore been suggested as a versatile model to study mechanisms of inflammation‐associated skeletal muscle loss.^80^

Limitations of both the TNBS and the DSS model include the irrelevance of the toxins themselves for the development of IBD in patients, and the resulting uncertainties regarding the significance of findings made in these models and the translation to the pathophysiology of the human disease.

The gut‐skeletal muscle axis has not been addressed in studies of experimental colitis. It has been shown that DSS colitis alters the expression of neurotrophins in smooth muscle cells^81^ and TNBS colitis leads to dysfunction of calcium channels in smooth muscle cells.^82^ Subsequently, gut dysmotility can lead to an impaired barrier function,^82^ which might contribute to muscle wasting. However, a link to skeletal muscle cells has not been firmly established yet.

### 3.3 Models of muscle wasting in pancreatic disorders

With a prevalence of approximately 17%, sarcopenia represents a frequent complication of CP.^27^ Muscle wasting in CP patients is closely associated with pancreatic exocrine insufficiency and has been linked to adverse health‐related outcomes.^27^, ^83^, ^84^ Acknowledging the high clinical relevance of chronic pancreatic disorders, several animal models for CP have been established. These include repetitive caerulein injections leading to recurrent acute pancreatitis, a well‐known cause for chronic pancreatitis.^85^ Caerulein is a cholecystokinin analogue which causes acute pancreatitis when given in supraphysiological concentrations,^86^ which is mediated by the balance between trypsin‐activating and trypsin‐degrading lysosomal enzymes.^87^, ^88^ The caerulein model can be modified experimentally ranging from mild oedematous, to necrotizing and to chronic pancreatitis.^89^ Intraperitoneal injections over 10 weeks twice per week lead to repetitive bouts of acute pancreatitis and ultimately leading to chronic injury of the organ. Another approach is based on an obstruction of the pancreatic duct that is performed by a ligation followed by a single caerulein injection, leading to severe necrotizing pancreatitis and subsequent fibrosis.^90^ This model is also characterized by a loss of digestive enzyme production in the pancreas. A third entity of experimental models comprises genetically engineered mice. Mice with a heterozygous p.D23A mutation in the cationic trypsinogen isoform T7 show a 50‐fold increase of trypsin activity that subsequently leads to acute pancreatitis. Starting at 4 weeks of age first signs of chronic disease occur with diffuse interstitial fibrosis, fatty tissue replacement and organ atrophy. These mice suffer from a significant weight loss.^91^ Notably, homozygous mice become stunted and die with an age of 2 months. Mice with a knock‐in of the p.K24R mutation developed only a fivefold increase of trypsinogen autoactivation and pancreatitis only with additional caerulein injections.^92^

Given the high clinical significance, it comes as a surprise that mechanistic studies employing animal models of muscle wasting in CP are largely missing. Instead, sarcopenia in another pancreatic disease has drawn the attention of experimentalists: approximately 80% of patients with pancreatic ductal adenocarcinoma (PDA) suffer from malnutrition, constituting the highest rate of cachexia in all human malignancies. The most prominent pathophysiologic anomaly in these cachectic patients is wasting of skeletal muscle.^93^

Pancreatic ductal adenocarcinoma‐associated cachexia has been reproduced in different animal models of the disease, including heterotopic and orthotopic tumour transplantation models and genetically engineered mouse models (GEMM). Studies in these models have identified some essential mediators of muscle wasting in the context of PDA and point to a distinguished and non‐redundant role of members of the TGF‐β protein family. Specifically, blockade of TGF‐β itself was shown to decrease the metabolic changes associated with cancer cachexia and improve overall survival of tumour‐bearing mice.^94^ Furthermore, systemic blockade of activin signalling could preserve muscle and also prolonged survival, while skeletal muscle‐specific activin blockade was only protective for weight loss.^95^

Recent studies by Yang et al^96^ implicate the zinc transporter ZIP4 not only into growth of orthotopic pancreatic tumours in mice, but also specifically into loss of muscle mass. Noteworthy, signalling through myeloid differentiation factor (MyD88), a key component of the innate immune system, was also found to be essential in the development of cachexia in PDA.^97^ The role of the muscle microenvironment in the promotion of PDA‐associated cachexia was highlighted by He et al,^98^ who identified NF‐κB‐mediated *pax7* dysregulation in myogenic progenitor cells as a key mechanism.

To this end, no model of experimental PC fully recapitulates malnutrition and muscle wasting as they occur in the context of the human disease. Thus, tumour‐bearing mice frequently develop lethal wasting within days or weeks, while PDA patients are likely to experience asymptomatic alterations long before the onset of overt wasting. In addition, specific limitations of the individual models (eg use of immunodeficient mice, artificial microenvironment of tumour growth, or presence of mutations in all cells of the pancreas) have to be taken into account.

Recently, Talbert et al have introduced an advanced GEMM of PDA‐associated sarcopenia, termed KPP mouse: these mice exhibit the genotype *Kras^+/G12D^*, *Ptf1a^+/ER‐Cre^*, *Pten^f/f^* and express Cre recombinase in a tamoxifen‐dependent manner. Upon tamoxifen treatment, Cre simultaneously inactivates two (floxed) alleles of the tumour suppressor *Pten* and induces mutant *Kras*. The new model allows PDA and its associated cachexia to be induced only after ending of neonatal growth of mice, creating a condition that more accurately reflects the actual loss of muscle (and adipose) tissue in patients with PDA.^99^ The contribution of exocrine insufficiency to the development of PDA‐associated muscle wasting in KPP mice has not been addressed yet. Given that cachexia of PDA patients is enhanced by the lack of digestive enzymes,^84^ this aspect needs to be studied further. In the absence of specific therapies for cancer‐associated sarcopenia, it also remains an open question which mouse model(s) are most suitable to evaluate experimental treatments. While GEMM offer the advantage of endogenous tumour development, patient‐derived xenografted PDA may more closely resemble the characteristics of the individual tumour. Employing both PDA xenografts and GEMM in parallel, Henderson et al gained evidence for an anti‐sarcopenic effect of the histone deacetylase inhibitor AR‐42, paving the road for follow‐up studies with a similar experimental design.^100^

**Figure 1:**
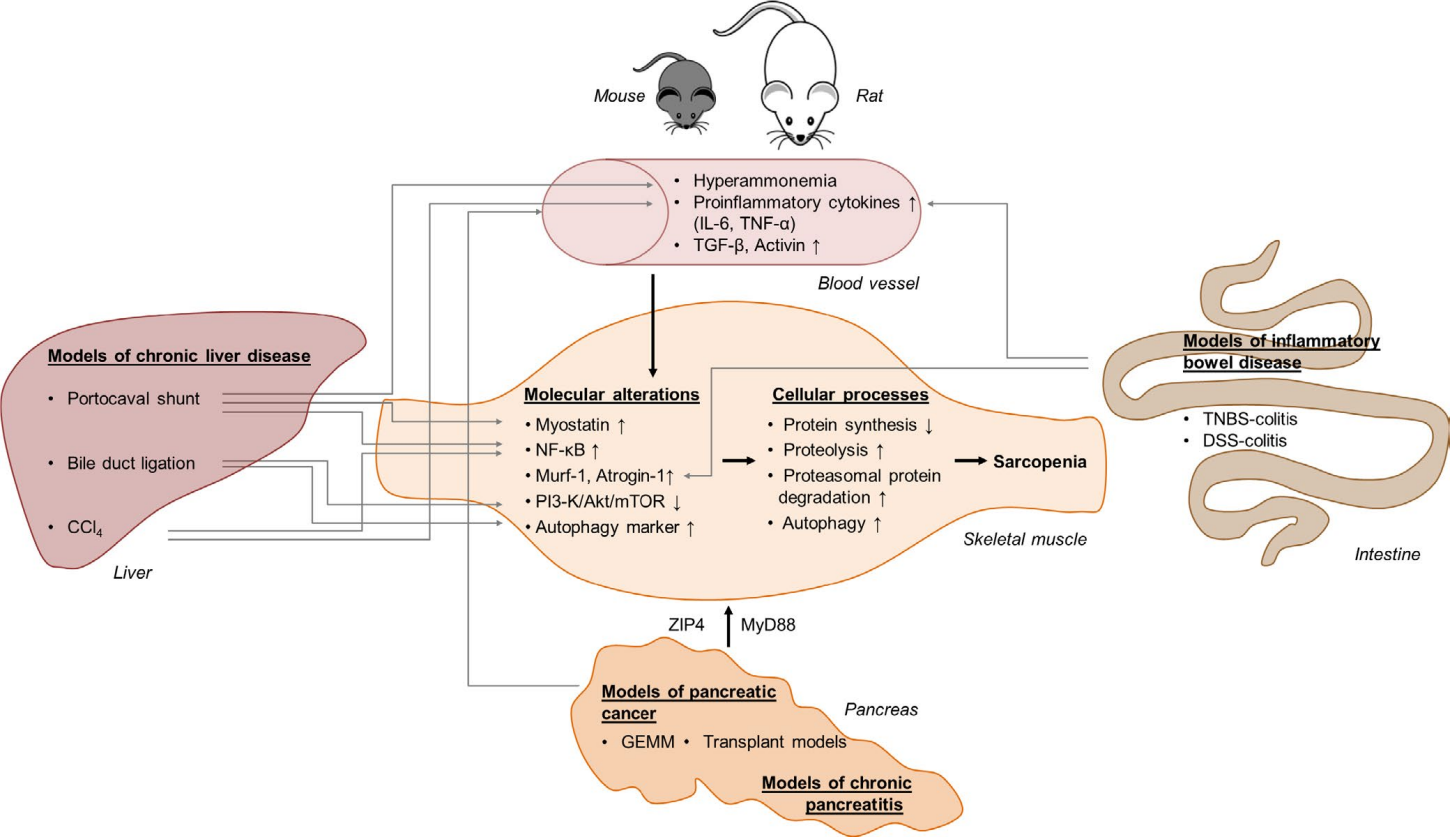
Mechanisms of sarcopenia addressed by animal models of gastrointestinal diseases. Rodent models of chronic liver diseases, inflammatory bowel diseases and pancreatic disorders including cancer induce sarcopenia through a variety of molecular and cellular processes. Increased transcription of *myostatin*, *Murf‐1* and/or *atrogin‐1* leads to an increased protein degradation (mostly via proteasomal degradation) and decreased protein synthesis. Additionally, NF‐κB can trigger *myostatin*, *Murf‐1* and *atrogin‐1*. Myostatin furthermore inhibits PI 3‐K/Akt/mTOR signalling, which in turn leads to autophagy. Molecular processes can also be influenced by hyperammonemia or cytokines such as IL‐6, TNF‐α, TGF‐β or activin, which circulate in the blood system. Especially hyperammonemia and the pro‐inflammatory cytokines IL‐6 and TNF‐α stimulate autophagy and NF‐κB‐dependent protein degradation. Models of chronic liver disease (portocaval shunt, bile duct ligation, CCl_4_), inflammatory bowel disease (TNBS and DSS colitis), chronic pancreatitis and pancreatic cancer (GEMM, transplant models) address different aspects of the complex system that can lead to sarcopenia. CCl_4_, carbon tetrachloride; DSS, dextran sulphate sodium; GEMM, genetically engineered mouse models; IL‐6, interleukin 6; MyD88, myeloid differentiation factor; Murf‐1, muscle RING‐finger protein‐1; NF‐κB, nuclear factor ‘kappa‐light‐chain‐enhancer’ of activated B cells; PI3‐K/Akt/mTOR, phosphatidylinositol 3‐kinase/protein kinase B/mammalian target of rapamycin; TGF‐β, transforming growth factor‐β; TNBS, trinitrobenzene sulphonic acid; TNF‐α, tumour necrosis factor‐α; ZIP4, zinc transporter

Figure 1 summarizes mechanisms of sarcopenia and their reflection by animal models of gastrointestinal diseases.

## 4 EXPERIMENTAL MODELS—FUTURE DIRECTIONS

Recent advancements in the field of omics technologies have also opened the avenue for novel approaches to elucidate the gut‐skeletal muscle axis in chronic gastrointestinal disorders with muscle wasting. Here, animal models may supplement studies in patients because of their specific advantages, including simplified standardization, the possibility to monitor the entire course of the disease from a defined starting point up to the endpoint of the study and the ability to analyse gastrointestinal and muscle tissue. Proteomics and metabolomics technologies have reached a technical state where they may provide a comprehensive overview of proteins and metabolic markers in gastrointestinal organs, plasma and skeletal muscles that may be mechanistically involved in disease‐associated muscle wasting. The emerging role of gut microbiota as sources of metabolites influencing muscle trophism and as key modulators of intestinal barrier function needs to be addressed further by microbiome‐involving studies. Employment of such unbiased approaches will enable hypothesis‐driven follow‐up investigations that will eventually decipher drivers and key mediators of sarcopenia and also help to identify novel therapeutic targets.

With respect to the animal models themselves, significant progress can be expected from the accelerated generation of GEMM by the CRISPR/Cas9 technology. Mice with modified genetic backgrounds will be increasingly used not only in studies on cancer‐associated sarcopenia, but also in the context of non‐malignant gastrointestinal diseases with muscle wasting.

To this end, studies employing animals as models for the experimental treatment of malnutrition and sarcopenia in association with gastrointestinal diseases are scarce. Moreover, they were largely restricted to the analysis of effects on functions of individual organs, such as muscle, liver, small or large intestine. Important candidates for follow‐up studies in appropriate animal models are gastrointestinal hormones with potential anti‐sarcopenic effects, including IGF‐1, leptin, ghrelin and GLP‐1. Other promising approaches may target mediators of systemic inflammation and molecular triggers of sarcopenia. Of course, the long‐term success of all such approaches depends on, and is limited by, the control of the underlying primary gastrointestinal disease itself.

## 5 CONCLUSIONS

In chronic gastrointestinal diseases such as LC, IBD, CP and PC, an incomplete pathophysiological understanding of the gut‐skeletal muscle axis hampers the development of specific anti‐sarcopenic therapies. Rodent models are useful to gain further mechanistic insights into disease‐associated muscle wasting and to identify promising molecular targets for therapeutic interventions. Since none of the established animal models completely resembles the human disease, care needs to be taken when choosing the appropriate model to address dedicated scientific questions. Further progress in the field can be expected from recent advancements of omics technologies (eg for microbiome and metabolome studies), and from the rapidly increasing availability of well‐defined genetically engineered mouse models.

## CONFLICT OF INTEREST

None to declare.

## AUTHOR CONTRIBUTIONS

Luise Ehlers, Karen Bannert and Robert Jaster conceived and drafted the manuscript. Luise Ehlers, Karen Bannert, Sarah Rohde, Peggy Berlin, Johannes Reiner, Mats Wiese, Julia Doller, Markus M. Lerch, Ali A. Aghdassi, Fatuma Meyer, Luzia Valentini, Ottavia Agrifoglio, Cornelia C. Metges, Georg Lamprecht and Robert Jaster contributed with critical intellectual input, read and revised the final draft.
